# *Escherichia coli* Urinary Tract Infections from a Romanian Pediatric Hospital: Antimicrobial Resistance Trends, ESBL Prevalence, and Empirical Treatment Implications

**DOI:** 10.3390/antibiotics14090855

**Published:** 2025-08-24

**Authors:** Daniela Păcurar, Alexandru Dinulescu, Andrei-Vlad Totu, Irina Dijmărescu, Mirela-Luminița Pavelescu

**Affiliations:** 1 Faculty of Medicine, Department of Pediatrics, “Carol Davila” University of Medicine and Pharmacy, 050474 Bucharest, Romania; daniela.pacurar@umfcd.ro (D.P.); andrei-vlad.totu0125@rez.umfcd.ro (A.-V.T.); mirela.pavelescu@umfcd.ro (M.-L.P.); 2 Department of Pediatrics, Emergency Hospital for Children “Grigore Alexandrescu”, 011743 Bucharest, Romania

**Keywords:** *Escherichia coli*, urinary tract infection, pediatric, antimicrobial resistance, ESBL, empirical treatment, Romania, fosfomycin, nitrofurantoin, carbapenems

## Abstract

Background: Urinary tract infections (UTIs) are among the most common bacterial infections in children, with *Escherichia coli* as the leading pathogen. The rise in antimicrobial resistance, particularly extended-spectrum β-lactamase (ESBL) production, complicates empirical therapy, especially in countries with limited surveillance like Romania. Methods: We conducted a retrospective study on 248 pediatric patients aged 0–17 years diagnosed with *E. coli* UTI and admitted to a children hospital from Bucharest, Romania, between 2022 and 2024. Data collected included clinical presentation, laboratory values, and antimicrobial susceptibility testing. Patients were divided into ESBL and non-ESBL groups, and statistical comparisons were performed using SPSS v25. Results: Infants accounted for 68.1% of cases, with male sex predominating in this group (85.3%). ESBL-producing strains were identified in 19% of patients, more frequently in males (25% vs. 13.6%, *p* = 0.024). While inflammatory markers (CRP, leukocytes, neutrophils) were higher in complicated infections, they were paradoxically lower in ESBL cases. Non-ESBL isolates were highly susceptible to fosfomycin, third-generation cephalosporins, nitrofurantoin, and gentamicin. ESBL isolates showed resistance to most β-lactams but retained high susceptibility to fosfomycin (100%), carbapenems (>89%), cefoxitin (89.4%), and amikacin (85.1%). Conclusions: Our findings support the use of fosfomycin, nitrofurantoin, and selected aminoglycosides as viable empirical options in pediatric UTIs. Given the substantial ESBL prevalence and resistance to commonly used oral agents, local antibiogram data should guide empirical treatment strategies to preserve antibiotic efficacy and combat resistance.

## 1. Introduction

Urinary tract infections (UTIs) are among the most common bacterial infections in children and constitute a frequent reason for medical evaluation, hospital admission, and antibiotic prescription [1,2,3,4]. They are especially important in infants and young children, in whom symptoms may be nonspecific and diagnosis can be often delayed [2,5,6]. Early and effective treatment is essential, as UTIs, particularly in neonates and young infants, can lead to renal scarring, hypertension, or chronic kidney disease, if left untreated, and can also be a source of serious systemic infections (sepsis) or local infections (perinephric abscess) [5,6,7,8]. The global prevalence of UTIs in children is approximately 8%, ranging from 3–5% in female patients to 1% in male patients [6,9,10].

According to the European Association of Urology (EAU), UTIs are classified by localization as systemic UTIs (acute pyelonephritis) and localized UTIs (acute cystitis) [11]. The first diagnosed UTI is classified as a first episode, requiring careful evaluation to determine the need for imaging and follow-up, while recurrent UTIs are defined by the presence of multiple episodes over time (at least three infections in 1 year or two in the last 6 months) and may suggest an underlying condition. Relapse is defined as a recurrence with the same micro-organism within 2 weeks of completing therapy, whilst a reinfection is defined as a UTI that occurs in more than 2 weeks after treatment [12,13,14]. UTIs can also be classified as complicated and uncomplicated. Uncomplicated UTIs occur in otherwise healthy children with no structural or functional abnormalities of the urinary tract, while complicated UTIs are associated with underlying anatomical or functional anomalies, urinary catheterization, or systemic signs of infection. This classification is essential for guiding both the diagnostic approach and therapeutic decisions [15,16,17].

*Escherichia coli (E. coli)* remains the primary etiologic agent of UTIs in children, responsible for approximately 80–90% of community-acquired cases [6,18]. Its pathogenicity is driven by a variety of virulence factors, such as adhesins (e.g., P fimbriae), siderophores, and toxins, which facilitate colonization and invasion of the urinary tract [19,20,21]. In recent years, rising antimicrobial resistance among *E. coli* strains, especially the emergence of extended-spectrum β-lactamase (ESBL) producers, has made empirical treatment increasingly difficult [22,23,24]. Resistance to commonly used antibiotics, such as ampicillin, trimethoprim-sulfamethoxazole, and even third-generation cephalosporins, has been documented with increasing frequency [25,26,27]. Carbapenems are usually recommended by international data in cases of ESBL-producing *E. coli* strains, and, in our country, the guide elaborated by the National Institute of Infectious Diseases Prof. Dr. Matei Balș supports this recommendation [28,29,30].

The global spread of multidrug-resistant *E. coli* poses a particular challenge in countries where antibiotic stewardship is still developing. In Romania, regional surveillance data is scarce, and empirical treatment decisions are often made in the absence of local resistance profiles. This retrospective study aims to assess the antimicrobial susceptibility of *E. coli* strains isolated from pediatric UTI cases in a Romanian tertiary pediatric hospital over a three-year period (2022–2024) in order to contribute to evidence-based treatment strategies and support national efforts toward rational antibiotic use in children.

## 2. Results

### 2.1. Clinical Characteristics of E. coli Pediatric UTIs

We found 248 *E. coli* isolates that met the inclusion criteria. UTIs were slightly more frequent in the 132 (53.2%) female patients. The age of the patients was not normally distributed (*p* < 0.001), with a median of 5 months (2–23.75). Most patients were infants, totaling 169 (68.1%). We report a lower median for age in males, 3 months (1–6.75), when compared to females, 9 months (4–50.25) (*p* < 0.001).

The majority of the UTIs were acute pyelonephritis (76.6%). Most of the UTIs were first episodes (83.9%), followed by recurrent UTIs (13.7%) and relapses (2%). Sixty-seven (27%) were complicated UTIs. Urinary tract malformations were present in 49 patients (19.8%). Additionally, 23 patients (9.3%) were malnourished, 12 (4.8%) had obesity, and 4 (1.6%) were immunocompromised, with two being due to neuromuscular diseases, one with stage 4 chronic kidney disease, and one receiving immunosuppressive therapy for inflammatory bowel disease, while six patients (2.4%) had chronic constipation. There was only 1 (0.4%) case of nosocomial UTI.

The complete descriptive statistics of the study group is presented in Table 1.

Considering the age group, in infants, the proportion of male patients was significantly higher, 99 (85.3%) vs. 70 (53%) in female patients (*p* < 0.001). All males were uncircumcised.

From all patients with recurrent infections, 55.9% were in complicated UTIs, and all patients with reinfections were in the same group (complicated UTIs). Primary infections were more frequently diagnosed in uncomplicated cases (77.4%). The difference was statistically significant (*p* < 0.001) (Table 2).

**Figure 1:**
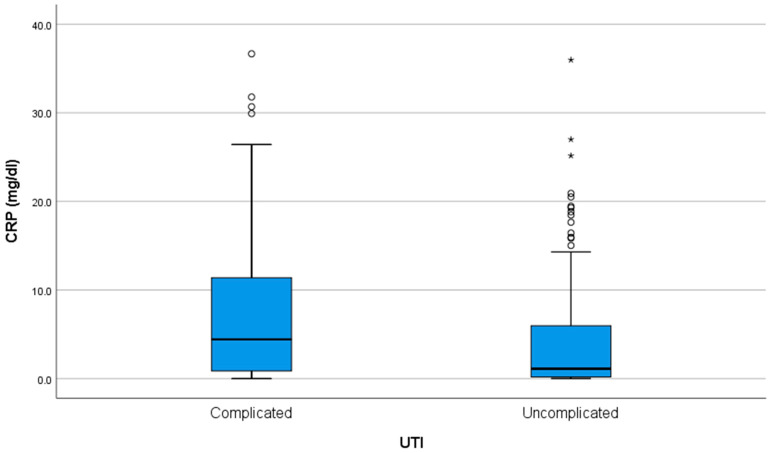
CRP levels at admission in children with complicated versus uncomplicated urinary tract infections (UTIs). The interior bars indicate the medians while the whiskers extend to the maximum and minimum of the data; ◦ = outlier; * = far outlier.

The C-reactive protein (CRP) had a median of 1.58 (0.26–7.79) mg/dL (normal = 0–0.5 md/dL). There was a weak (R = 0.323) but statistically significant (*p* < 0.001) positive correlation between CRP levels and age, with older patients tending to have slightly higher CRP values. There was no association between the CRP level and the episode number (*p* = 0.407). The CRP level was significantly higher in complicated UTIs, at 4.42 (0.86–11.46) vs. 1.11 (0.16–6.15) when compared to uncomplicated cases (*p* < 0.001) (Figure 1).

Leukocyte number had a median of 13.3 (10.16–18.56) × 10^9^/L. There was no significant correlation between leukocyte count and age (*p* = 0.714). There was no association between leukocyte count and the episode number (*p* = 0.241) or type of UTI, complicated or uncomplicated (*p* = 0.327).

**Figure 2:**
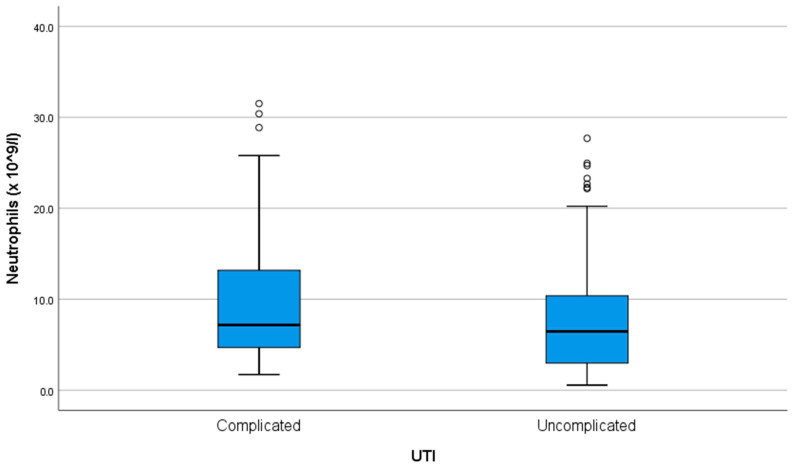
Neutrophil count at admission in complicated versus uncomplicated UTIs. ◦ = outlier.

Neutrophil levels had a median of 6.64 (3.8–11.87) × 10^9^/L. There was a moderate (R = 0.665) and statistically significant (*p* < 0.001) positive correlation between age and neutrophil count, with older patients tending to have higher neutrophil levels. There was no association between neutrophil count and the number of episodes (*p* = 0.935). Neutrophil count was higher in complicated UTIs, at 7.16 (4.63–13.27) vs. 6.46 (2.94–10.42) in uncomplicated ones (*p* < 0.001) (Figure 2).

On urinalysis, 55 (22.2%) patients had positive nitrites. The group with positive nitrites was older, at 11 (3–60) months when compared to the group with negative nitrites, at 4 (2–13.5) months (*p* = 0.004). There was no difference in nitrites positivity rate between the complicated and uncomplicated groups (*p* = 1.00). There was no association between positive nitrites and CRP level (*p* = 0.797), leukocyte count (*p* = 0.406), or neutrophil count (*p* = 0.980). Fifty-eight (23.4%) isolates met the criteria for MDR.

We found 47 (19%) ESBL-producing *E. coli*. There was a statistically significant association between sex and ESBL positivity (*p* = 0.023), with males having a significantly higher proportion of ESBL-positive cases (25%) compared to females (13.6%). CRP (*p* = 0.003), leukocyte (*p* = 0.025), and neutrophil (*p* = 0.010) count were significantly higher in the non-ESBL-producing group.

Age (*p* = 0.85), UTI localization (*p* = 0.448), type of UTI (complicated/uncomplicated (*p* = 0.859)), recurrence (*p* = 0.938), or positivity rate of nitrites (*p* = 0.117) were not significantly different between ESBL-producing and non-ESBL-producing *E. coli* groups. ESBL-producing isolates were much more likely to exhibit MDR compared to non-ESBL producers (*p* < 0.001).

The extensive comparison between the two groups can be found in Table 3.

### 2.2. Antibiotic Susceptility Patterns of E. coli from Pediatric UTIs

**Figure 3:**
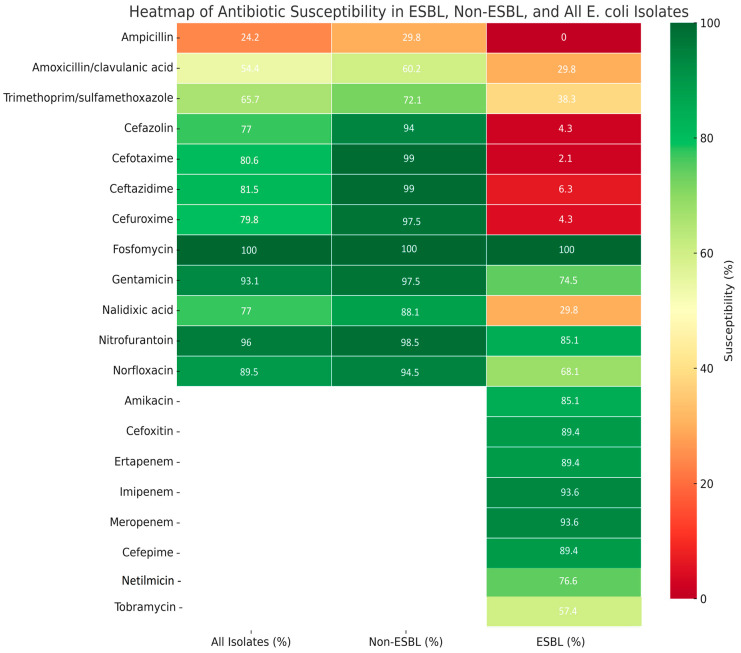
Overall antibiotic susceptibility of *E. coli* isolates (*n* = 248) from pediatric urinary tract infections.

When analyzing both groups combined, fosfomycin remained the most effective agent, with 100% susceptibility across all isolates. Nitrofurantoin and gentamicin also showed excellent activity, with susceptibility rates of 96% and 93.1%, respectively. Ampicillin demonstrated poor performance, with only 24.2% of isolates susceptible. Amoxicillin/clavulanic acid and trimethoprim/sulfamethoxazole showed moderate effectiveness. These findings reinforce the role of fosfomycin, nitrofurantoin, and selected aminoglycosides as reliable treatment options, especially in the context of rising beta-lactam resistance (Figure 3).

In the non-ESBL-producing group, most antibiotics showed high efficacy. Fosfomycin demonstrated universal effectiveness (100%), followed closely by third-generation cephalosporins (cefotaxime, ceftazidime—99%), nitrofurantoin (98.5%), and gentamicin (97.5%). In contrast, ampicillin was largely ineffective, with only 29.8% of isolates susceptible. Amoxicillin/clavulanic acid and trimethoprim/sulfamethoxazole showed moderate activity, with efficacy rates of 60.2% and 72.1%, respectively.

Overall, the non-ESBL-producing isolates showed high susceptibility to fosfomycin, third-generation cephalosporins, nitrofurantoin, and aminoglycosides, while ampicillin displayed limited efficacy.

In the ESBL-producing group, resistance to beta-lactams was widespread. All isolates were resistant to ampicillin, and 89.4–97.9% were resistant to first- and third-generation cephalosporins. Amoxicillin/clavulanic acid and trimethoprim/sulfamethoxazole were also largely ineffective; fosfomycin retained full activity (100% susceptible). High susceptibility was also observed with carbapenems (imipenem and meropenem: 93.6% each) and selected aminoglycosides, particularly amikacin (85.1%). Cefoxitin and nitrofurantoin showed good activity, with efficacy rates of 89.4% and 85.1%, respectively

ESBL-producing *E. coli* isolates demonstrated high resistance to third-generation cephalosporins, including cefotaxime (97.9%) and ceftazidime (89.4%), in contrast to the non-ESBL-producing group, which exhibited very high susceptibility to these agents (99% each). In addition to beta-lactams, resistance was notably higher in the ESBL group for amoxicillin/clavulanic acid (29.8% susceptible vs. 60.2%) and trimethoprim/sulfamethoxazole (38.3% vs. 72.1%), while agents such as fosfomycin, nitrofurantoin, and carbapenems retained excellent activity in both groups.

To illustrate a comparison, susceptibility rates between ESBL-producing and non-ESBL-producing *E. coli* isolates are summarized in Table 4.

## 3. Discussion

This study provides a detailed analysis of the antimicrobial resistance patterns of *E. coli* in pediatric UTIs in a tertiary hospital from Romania, emphasizing the comparison between ESBL-producing and non-ESBL-producing strains. Most of the patients (68.1%) included in this study were infants, a finding that is compatible with the literature, where the highest prevalence of UTI in children is described in uncircumcised boys under 3 months old and girls under 1 year old [31]. The predominance of male gender in infants found in this study (85.3% vs. 53%, *p* < 0.001) is consistent with the literature, where male predominance is observed in early infancy, especially in uncircumcised males, while UTIs become more common in females as children grow older [31,32,33,34,35]. All males in this study were uncircumcised, with circumcision being an extremely rare practice in this country [36].

One-fifth (19.8%) of the cases had urinary tract malformations, reinforcing the EAU guideline that children with a first febrile UTI should undergo renal and urinary tract ultrasound, as abnormal findings are observed in about 15% of cases. [35]. As expected, recurrent infections were more frequently observed among complicated cases, with more than half of the recurrent episodes (55.9%) being classified as complicated, in contrast to primary infections, which were predominantly uncomplicated (77.4%), reinforcing the need for further urological evaluation in such cases [15,35]. Complicated cases also displayed higher inflammatory markers (CRP and neutrophil count) (*p* < 0.005), suggesting more severe disease presentations [37,38]. On the urinalysis, 55 (22.2%) patients had positive nitrites; they were not associated with the presence of complicated infection or higher inflammatory parameters, and we did not find any studies that analyzed this idea.

Regarding ESBL-producing prevalence, our findings show that 19% of isolates were ESBL-producing, with a statistically significant higher prevalence in male patients (25% vs. 13.6% in females) (*p* = 0.024). This trend—6.4% male vs. 5.2% female (*p* < 0.001)—was also reported in a study conducted by Khanal et al. in 2024 on adult subjects, including 85844 *E. coli* urine samples [39]. While ESBL and non-ESBL-producing infections did not differ in terms of age, localization, or complexity of infection, the inflammatory response (CRP, leukocyte, and neutrophil counts) was significantly lower in ESBL cases, a result that warrants further investigation.

The antimicrobial sensitivity profile revealed critical differences between the two groups. In non-ESBL-producing strains, third-generation cephalosporins, fosfomycin, nitrofurantoin, and aminoglycosides maintained excellent efficacy. Ampicillin, as expected, showed poor activity, reflecting its limited current role in empirical treatment [1]. In contrast, ESBL-producing strains exhibited broad resistance to beta-lactams, including ampicillin, amoxicillin/clavulanic acid, and most cephalosporins, with cefoxitin being a notable exception (89.4% susceptible). Carbapenems, including imipenem, meropenem, and ertapenem, retained high efficacy (>89%), consistent with their established role as last-line agents in ESBL-positive infections [40,41,42]. Fosfomycin, again, showed 100% effectiveness, reinforcing its value as an oral treatment option [43]. Among aminoglycosides, amikacin and netilmicin remained effective in the majority of cases, whereas tobramycin had lower activity. Amoxicillin/clavulanic acid showed suboptimal efficacy in both groups, especially in ESBL-producing strains where resistance and intermediate susceptibility reached a combined 68.1%. These findings highlight the urgent need to re-evaluate empirical therapy recommendations, particularly in areas with known ESBL-producing *E. coli* high prevalence.

Our findings align closely with previously published studies on pediatric UTIs from Romania. Miron et al. (2021) showed low susceptibility rates of *E. coli* to ampicillin (25.6%) and amoxicillin/clavulanate (37.2%) [44]. Approximately one-third (27%) of their isolates were resistant to trimethoprim/sulfamethoxazole [44]. We observed similarly low susceptibility rates in our non-ESBL group (ampicillin 29.8%, amoxicillin/clavulanate 60.2%, trimethoprim/sulfamethoxazole 72.1%) and even lower efficacy among ESBL producers. Duicu et al. (2021) reported similar trends for those three aforementioned antibiotics [45]. These Romanian trends are also seen in adults. Borcan et al. (2024) published a study of 3131 *E. coli* positive urine cultures for which they report a low susceptibility to ampicillin (41%) and trimethoprim/sulfamethoxazole (68%) and high susceptibility to fosfomycin (97%) and nitrofurantoin (98%) [46]. In adult populations, Chibelean et al. (2020) reported in a two-center study, including 2763 *E. coli* isolates from adult males, a high susceptibility to amikacin (91.7%), meropenem (97.1%), and fosfomycin (86.6%), while lower rates were observed for amoxicillin/clavulanate (72%) and levofloxacin (62.3%) [47]. Similarly, Mares et al. (2023), analyzing 613 *E. coli* isolates from adult females, found high susceptibility to fosfomycin (92.3%) and carbapenems (90.3–95.3%) [48]. However, in contrast to our findings, they reported a markedly lower susceptibility to nitrofurantoin (65.5%).

Studies from other countries report a consistent pattern: elevated resistance to penicillin, cephalosporins, and trimethoprim/sulfamethoxazole, contrasted by preserved susceptibility to nitrofurantoin, amikacin, carbapenems, and fosfomycin [33,49,50,51]. Bagnasco et al. (2025) described 72.1% resistance to amoxicillin-clavulanate and 75.6% to cotrimoxazole and a very high susceptibility to nitrofurantoin (99.8%) and fofomycin (99.3%) in 1324 *E. coli* isolates from Italian infants under 6 months of age [33]. Another study conducted by Mahajan et al. (2024) that analyzed 3511 *E. coli* isolates from USA children between 2014–2023 showed high susceptibility to carbapenems (99.9–100%) and nitrofurantoin (98.1%) and poor efficacy of trimethoprim/sulfamethoxazole (69.7%) [50]. Data from Poland were made available by Kawalec et al. (2023), who evaluated 165 pediatric *E. coli* isolates and reported 68% sensitivity for amoxicillin-clavulanate, with 76.7% for trimethoprim/sulfamethoxazole and 95.6% for nitrofurantoin [51]. Abdelgalil et al. (2024) conducted a study in Saudi Arabia, which analyzed 50 children aged 0–14 years with urine cultures positive for ESBL-producing *E.coli,* and reported a susceptibility rate of 40% to amoxicillin-clavulanate, with 36% to trimethoprim/sulfamethoxazole and 78% to gentamicin, as opposed to a high susceptibility rate to nitrofurantoin (92%) and amikacin (98%), while all isolates were sensitive to carbapenems (meropenem and imipenem) [49]. A multicenter study, published by Ny et al. (2019), involving outpatient women with uncomplicated UTIs across six Northern and Eastern European countries (Finland, Germany, Latvia, Poland, Russia, and Sweden), found very low resistance to nitrofurantoin (1.2%) and fosfomycin (1.3%) while resistance to ampicillin, trimethoprim-sulfamethoxazole, and amoxicillin/clavulanic acid ranged from 17% to 40%. Notably, none of the isolates were resistant to meropenem, aligning with our findings of excellent susceptibility to fosfomycin and carbapenems in pediatric ESBL and non-ESBL strains [52]. A recent World Health Organization (WHO)/ European Centre for Disease Prevention and Control (ECDC) executive summary, utilizing data from the EARS-Net and CAESAR networks, highlights that, in 2023, the estimated incidence of bloodstream infections caused by *E. coli* resistant to third-generation cephalosporins was 10.35 per 100,000 population across the EU/EEA, slightly exceeding the EU target of 9.67 per 100,000. This underscores how third-generation cephalosporin–resistant *E. coli* remains a persistent and high-priority public health challenge in Europe, aligning with the patterns observed in our pediatric cohort [53].

Consistent with national and international findings, our results confirm the poor activity of aminopenicillins and trimethoprim combinations in pediatric UTIs. However, fosfomycin, nitrofurantoin, and carbapenems remain extremely effective. ESBL-producing *E. coli* isolates showed the highest susceptibility rates to carbapenems (imipenem and meropenem: 93.6%) and cefoxitin (89.4%), indicating their retained efficacy. Global data confirm that amoxicillin/clavulanate and trimethoprim/sulfamethoxazole are among the most prescribed antibiotics, and in Romania, although we did not find data on the frequency of prescription for each antibiotic from our current practice, these antibiotics are frequently prescribed in children. However, our findings and those of other studies demonstrate these agents are losing usefulness due to resistance [54]. There are recommendations that empiric UTI treatment should be based on local resistance patterns, thus supporting updating Romanian empirical pediatric UTI protocols and other infections guidelines [55,56]. From our results, nitrofurantoin may be an excellent candidate for empiric antibiotic therapy in children with UTI from our country, especially since there is also an oral suspension form, and it has a good safety profile [57,58,59].

### Limitations

This study has several limitations. Its single-center retrospective design may limit the generalizability of the findings to other regions in Romania or countries with different antimicrobial resistance profiles. Molecular testing for specific resistance genes (e.g., *bla_CTX-M_*, *bla_SHV_*, *bla_TEM_*) was not performed, and thus the precise genetic mechanisms of ESBL production remain unidentified. We did not test for other β-lactamase types such as AmpC or carbapenemases, which may coexist and influence resistance patterns. Additionally, treatment regimens and clinical outcomes were not evaluated, limiting our ability to correlate in vitro resistance data with therapeutic success or failure.

## 4. Materials and Methods

### 4.1. Study Design and Setting

This was a retrospective cross-sectional study evaluating antibiotic susceptibility patterns in pediatric patients with *E. coli* UTIs admitted to the Pediatrics department of ‘’Grigore Alexandrescu” Emergency Hospital for Children, Bucharest, Romania from 1 January 2022 to 31 December 2024. The study was conducted at one of the largest pediatric hospitals in Romania, located in the capital city, Bucharest, with 120 beds in the Pediatrics Department and approximately 7000 admissions per year; together with three other pediatric hospitals and several general hospitals with pediatric wards (e.g., infectious diseases and oncology), it serves the capital’s pediatric population of around 300,000 children, while nearly half of the admitted cases come from outside Bucharest, underscoring its role as a national tertiary referral center.

### 4.2. Study Population and Inclusion Criteria

We included in the study group children aged 0 to 17 years diagnosed with UTI (pyelonephritis or cystitis) with *E. coli*. Patients with UTI of other etiologies or incomplete data were excluded.

During this time 20,726 urine cultures were performed, and we identified 653 patients with UTIs admitted to the hospital during the established period. A number of 248 was selected to be further analyzed in this study, according to the inclusion and exclusion criteria.

### 4.3. Data Collection

For the selected patients, the following data were collected in Microsoft Excel from the hospital electronic register: data regarding epidemiology (age, gender, UTI type and risk factors) and laboratory test results (leukocyte count, neutrophil count, CRP, urinalysis, urine culture, and antimicrobial testing).

### 4.4. Definitions

- Positive urine culture: growth of ≥10^5^ CFU/mL of a single uropathogen in a properly collected urine sample.
- Pyelonephritis: fever ≥38.5 °C, flank/abdominal pain, high inflammatory markers (leukocyte count, CRP) and positive urine culture.
- Cystitis: symptoms suggestive for a lower urinary tract infection (dysuria, frequency, and urgency), no fever, normal CBC and inflammatory markers (C reactive protein/ESR/Fibrinogen) and a positive urine culture.
- Multidrug-resistant (MDR): resistance to ≥1 agent in ≥3 antimicrobial classes.

### 4.5. Laboratory Methods

Urine samples were collected using sterile containers. *Escherichia coli* isolates were identified and tested in accordance with Clinical and Laboratory Standards Institute (CLSI) guidelines. Bacterial identification was initially performed using conventional biochemical tests and confirmed by matrix-assisted laser desorption/ionization time-of-flight mass spectrometry (MALDI-TOF MS). Biochemical and enzymatic characterization was further validated using the VITEK automated card system (bioMérieux).

### 4.6. Antibiotic Susceptibility Testing

The antibiotic susceptibility testing (AST) was performed in accordance with CLSI 2022–2024 standards [60]. Disc diffusion (Kirby–Bauer) was carried out on Mueller–Hinton agar (Oxoid) using antibiotic discs provided by ATB OXOID. Quality control was ensured using *E. coli ATCC 25922*. For MDR assessment, broth microdilution was performed using the Micronaut system (Merlin Diagnostika GmbH). Phenotypic screening for extended-spectrum β-lactamase (ESBL) production was performed for all isolates.

According to the antimicrobial testing of urine cultures, the patients were divided in two groups, non-ESBL-producing strains (N = 201) and ESBL-producing strains (N = 47).

Antibiotic sensitivity in non-ESBL urine culture was tested for the following antibiotics: ampicillin, amoxicillin/clavulanic acid, trimethoprim/sulfamethoxazole, cefazolin, cefotaxime, ceftazidime, cefuroxime, fosfomycin, gentamicin, nalidixic acid, nitrofurantoin, and norfloxacin. For the ESBL-producing group, previously stated antibiotics were tested, and additionally amikacin, cefoxitin, ertapenem, imipenem, meropenem, cefepime, netilmicin, and tobramycin were also tested.

Detailed AST and ESBL status for each isolate are available in Supplementary Table S1.

### 4.7. Statistical Analysis

The data were analyzed using IBM SPSS Statistics version 25 and illustrated using Microsoft Office Excel/Word 2013. Quantitative variables were tested for normal distribution using the Shapiro–Wilks test and were written as medians with interquartile ranges (IQR). Quantitative variables were tested between independent groups using Mann–Whitney U tests. The Kruskal–Wallis test was used to determine significant differences between two or more groups of an independent variable. Fisher’s exact test was used to determine the nonrandom associations between categorical variables with Bonferroni method used for correction. Spearman coefficient was used to search for correlations between non-normal distributed quantitative variables.

## 5. Conclusions

Pediatric UTIs caused by *E. coli* in our cohort showed a substantial burden of ESBL-producing strains, particularly in male infants. While most non-ESBL-producing strains remain susceptible to commonly used antibiotics, including third-generation cephalosporins, fosfomycin, and nitrofurantoin, ESBL-producing strains display high resistance to beta-lactams, including amoxicillin/clavulanic acid and cephalosporins. Fosfomycin, nitrofurantoin, and carbapenems demonstrated the highest efficacy across both groups and should be considered key agents in empirical and targeted treatment strategies. Given the increasing resistance patterns observed especially to commonly used oral agents, routine surveillance and updated local antibiograms are essential to guide effective empirical therapy and limit the spread of antimicrobial resistance in pediatric populations.

## Figures and Tables

**Table 1:** Descriptive statistics of population main characteristics.

Variable	N (%)	Median (IQR)
Year of admission		
2022	92 (37.1%)	
2023	78 (31.5%)	
2024	78 (31.5%)	
Gender		
Male	116 (46.8%)	
Female	132 (53.2%)	
Age (months)		5 (2–23.75)
Infant	169 (68.1%)	
>1 year old	79 (31.9%)	
UTI localization		
Acute pyelonephritis	190 (76.6%)	
Acute cystitis	58 (23.4%)	
UTI episode		
First episode	208 (83.9%)	
Recurrent	34 (13.7%)	
Relapse	5 (2%)	
Reinfection	1 (0.4%)	
Complicated UTI		
Yes	67 (27%)	
No	181 (73%)	
Patient history		
Urinary tract	49 (19.8%).	
malformations		
Immunocompromised	4 (1.6%)	
Malnutrition	23 (9.3%)	
Patients with obesity	12 (4.8%)	
Constipation	6 (2.4%)	

**Table 2:** Complicated vs. uncomplicated infection by type of UTI episode.

Complicated/Uncomplicated	Primary Infection (*n* = 208)	Relapse (*n* = 5)	Recurrent Infection (*n* = 34)	Reinfection (*n* = 1)	Reinfection (*n* = 1)
Complicated (*n* = 67)	**47 (22.6%)**	0	**19 (55.9%)**	1	0.001
Uncomplicated (*n* = 181)	**161 (77.4%)**	5	**15 (44.1%)**	0	

**Table 3:** Comparison between non-ESBL-producing and ESBL-producing *E.coli* groups.

Variable	Non-ESBL-Producing	ESBL-Producing	*p*
Number	201 (80%)	47 (19%)	
Age (months)	6 (2–28.5)	4 (2–9)	0.085
Sex			
Male	87 (75%)	**29 (25%)**	**0.024**
Female	114 (86.4%)	**18 (13.6%)**	
UTI localization			
Acute pyelonephritis	156 (82.1%)	34 (17.9%)	0.448
Acute cystitis	45 (77.6%)	13 (22.4%)	
Complicated UTI			
Yes	55 (82.1%)	12 (17.9%)	0.859
No	146 (80.7%)	35 (19.3%)	
UTI episode			
First episode	169 (81.3%)	39 (18.8%)	
Recurrent	27 (79.4%)	7 (20.6%0	
Relapse	4 (80%)	1 (20%)	**0.938**
Reinfection	1 (100%)	0 (0%)	
CRP (mg/dL)	**2.07 (0.37–8.72)**	**0.45 (0.10–2.33)**	**0.003**
Leukocytes (×10^9^/L)	**14.05 (10.39–19.15)**	**11.95 (9.51–14.20)**	**0.025**
Neutrophils (×10^9^/L)	**7.28 (3.95–12.82)**	**4.86 (3.05–8.07)**	**0.010**
Positive nitrites	49 (24.4%)	6 (12.8%)	0.117
MDR status	**17 (8.5%)**	**41 (87.2%)**	**0.001**

**Table 4:** Comparison of susceptibility rates between ESBL-producing and non-ESBL-producing *E. coli* isolates.

Antibiotic	Non-ESBL-Producing Isolates Susceptibility (%)	ESBL-Producing Isolates Susceptibility (%)	Fisher’s Exact Test (*p*)
Ampicillin	60 (29.8%)	0 (0%)	0.001
Amoxicillin/clavulanicacid	121 (60.2%)	15 (29.8%)	0.001
Trimethoprim/sulfamethoxazole	145 (72.1%)	18 (38.3%)	0.001
Cefazolin	189 (94%)	2 (4.3%)	0.001
Cefotaxime	199 (99%)	1 (2.1%)	0.001
Ceftazidime	199 (99%)	3 (6.3%)	0.001
Cefuroxime	196 (97.5%)	2 (4.3%)	0.001
Fosfomycin	201 (100%)	47 (100%)	-
Gentamicin	196 (97.5%)	35 (74.5%)	0.001
Nalidixicacid	177 (88.1%)	14 (29.8%)	0.001
Nitrofurantoin	198 (98.5%)	40 (85.1%)	0.001
Norfloxacin	190 (94.5%)	32 (68.1%)	0.001

## Data Availability

Data are contained within the article.

## Supplementary Materials

The following supporting information can be downloaded at: https://www.mdpi.com/article/10.3390/antibiotics14090855/s1, Table S1: Antibiotic susceptibility testing and ESBL status for each isolate. Table S2: Antibiotic sensitivity for both groups; Table S3: Antibiotic sensitivity for the non-ESBL-producing group; Table S4: Antibiotic sensitivity for the ESBL-producing group.

## Abbreviations

The following abbreviations are used in this manuscript:

UTIs	Urinary tract infections
*E. coli*	*Escherichia coli*
CRP	C-reactive protein
