# Supplementary material for: Escherichia coli Urinary Tract Infections from a Romanian Pediatric Hospital: Antimicrobial Resistance Trends, ESBL Prevalence, and Empirical Treatment Implications
Source: Antibiotics (Basel). 2025 Aug 24;14(9):855. doi: 10.3390/antibiotics14090855 (PMC12466793; doi:10.3390/antibiotics14090855)
Supplement: Supplementary file 1 [file antibiotics-14-00855-s001.zip › Supplementary Table S2.pdf]

Supplementary Table S2: Antibiotic sensitivity for both groups

Antibiotic	Susceptible, N (%)	Intermediate, N (%)	Resistant, N (%)
**Ampicillin**	60 (24.2%)	2 (0.8%)	186 (75%)
**Amoxicillin/clavulanic acid**	135 (54.4%)	59 (23.8%)	54 (21.8%)
**Trimethoprim/sulfamethoxazole**	163 (65.7%)	0 (0%)	85 (34.3%)
**Cefazolin**	191 (77%)	3 (1.2%)	54 (21.8%)
**Cefotaxime**	200 (80.6%)	0 (0%)	48 (19.4%)
**Ceftazidime**	202 (81.5%)	2 (0.8%)	44 (17.7%)
**Cefuroxime**	198 (79.8%)	1 (0.4%)	49 (19.8%)
**Fosfomycin**	248 (100%)	0 (0%)	0 (0%)
**Gentamicin**	231 (93.1%)	0 (0%)	17 (6.9%)
**Nalidixic acid**	191 (77%)	1 (0.4%)	56 (22.6%)
**Nitrofurantoin**	238 (96%)	3 (1.2%)	7 (2.8%)
**Norfloxacin**	222 (89.5%)	0 (0%)	26 (10.5%)
