# Supplementary material for: Escherichia coli Urinary Tract Infections from a Romanian Pediatric Hospital: Antimicrobial Resistance Trends, ESBL Prevalence, and Empirical Treatment Implications
Source: Antibiotics (Basel). 2025 Aug 24;14(9):855. doi: 10.3390/antibiotics14090855 (PMC12466793; doi:10.3390/antibiotics14090855)
Supplement: Supplementary file 1 [file antibiotics-14-00855-s001.zip › Supplementary Table S3.pdf]

Supplementary Table S3: Antibiotic sensitivity for the non-ESBL-producing group

Antibiotic	Susceptible, N (%)	Intermediate, N (%)	Resistant, N (%)
**Ampicillin**	60 (29.8%)	2 (1%)	139 (69.2%)
**Amoxicillin/clavulanic acid**	121 (60.2%)	41 (20.4%)	39 (19.4%)
**Trimethoprim/sulfamethoxazole**	145 (72.1%)	0 (0%0	56 (27.9%)
**Cefazolin**	189 (94%)	3 (1.5%)	9 (4.5%)
**Cefotaxime**	199 (99%)	0 (0%)	2 (1%)
**Ceftazidime**	199 (99%)	0 (0%)	2 (1%)
**Cefuroxime**	196 (97.5%)	1 (0.5%)	4 (2%)
**Fosfomycin**	201 (100%)	0 (0%)	0 (0%)
**Gentamicin**	196 (97.5%)	0 (0%)	5 (2.5%)
**Nalidixic acid**	177 (88.1%)	0 (0%)	24 (11.9%)
**Nitrofurantoin**	198 (98.5%)	0 (0%)	3 (1.5%)
**Norfloxacin**	190 (94.5%)	0 (0%)	11 (5.5%)
