# Supplementary material for: Escherichia coli Urinary Tract Infections from a Romanian Pediatric Hospital: Antimicrobial Resistance Trends, ESBL Prevalence, and Empirical Treatment Implications
Source: Antibiotics (Basel). 2025 Aug 24;14(9):855. doi: 10.3390/antibiotics14090855 (PMC12466793; doi:10.3390/antibiotics14090855)
Supplement: Supplementary file 1 [file antibiotics-14-00855-s001.zip › Supplementary Table S4.pdf]

Supplementary Table S4: Antibiotic sensitivity for the ESBL-producing group

Antibiotic	Susceptible, N (%)	Intermediate, N (%)	Resistant, N (%)
**Ampicillin**	0 (0%)	0 (0%)	47 (100%)
**Amoxicillin/clavulanic acid**	15 (29.8%)	18 (38.3%)	15 (31.9%)
**Trimethoprim/sulfamethoxazole**	18 (38.3%)	0 (0%0	29 (61.7%)
**Cefazolin**	2 (4.3%)	0 (0%)	45 (95.7%)
**Cefotaxime**	1 (2.1%)	0 (0%)	46 (97.9%)
**Ceftazidime**	3 (6.3%)	2 (4.3%)	42 (89.4%)
**Cefuroxime**	2 (4.3%)	0 (0%)	45 (95.7%)
**Fosfomycin**	47 (100%)	0 (0%)	0 (0%)
**Gentamicin**	35 (74.5%)	0 (0%)	12 (25.5%)
**Nalidixic acid**	14 (29.8%)	1 (2.1%)	32 (68.1%)
**Nitrofurantoin**	40 (85.1%)	3 (6.4%)	4 (8.5%)
**Norfloxacin**	32 (68.1%)	0 (0%)	15 (31.9%)
**Amikacin**	40 (85.1%)	1 (2.1%)	6 (12.8%)
**Cefoxitin**	42 (89.4%)	0 (0%)	5 (10.6%)
**Ertapenem**	42 (89.4%)	1 (2.1%)	4 (8.5%)
**Imipenem**	44 (93.6%)	0 (0%)	3 (6.4%)
**Meropenem**	44 (93.6%)	0 (0%)	3 (6.4%)
**Cefepime**	43 (89.4%)	1 (2.1%)	4 (8.5%)
**Netilmicin**	36 (76.6%)	2 (4.3%)	9 (19.1%)
**Tobramycin**	27 (57.4%)	1 (2.1%)	19 (40.5%)
